# Smart bacteria-responsive coatings for combating catheter-associated urinary tract infections

**DOI:** 10.1016/j.mtbio.2025.102191

**Published:** 2025-08-11

**Authors:** Xiaojin Liu, Marleen Kamperman

**Affiliations:** Polymer Science, Zernike Institute for Advanced Materials, University of Groningen, Nijenborgh 3, Groningen, 9747 AG, the Netherlands

**Keywords:** Urinary catheter, Stimuli-responsive coatings, Antibacterial materials, Theranostic coatings, CAUTI sensors

## Abstract

As one of the leading hospital-acquired infections (HAI), catheter-associated urinary tract infections (CAUTIs) can not only give rise to high treatment costs, but may also lead to serious after effects like pyelonephritis, sepsis, bacteremia, and even death. In order to reduce the infection risk, a lot of efforts have been devoted to the development of antibacterial urinary catheters such as antibiotic-coated catheters. With the emergence of antibiotic resistance, the design of alternative antibacterial strategies such as stimuli-responsive coatings for combating CAUTIs has attracted increasing interest in recent years. In this review, we summarize recent advances of bacteria-responsive coatings that are specially designed for urinary catheters. Based on the specific microenvironments of CAUTIs, various responsive coatings triggered by pH changes and bacterial metabolites such as enzymes and toxins, have been developed. The design principles, fabrication approaches and antibacterial performance of such smart coatings are discussed based on representative examples. Finally, a brief perspective is presented on the challenges and future of bacteria-responsive coatings for urinary catheters.

## Graphical abstract

**Figure:**
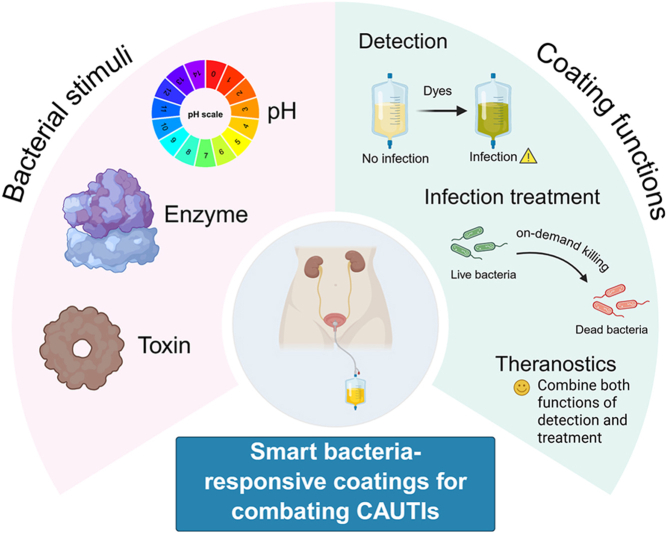

## 1 Introduction

### 1.1 Urinary catheters and catheter-associated urinary tract infections (CAUTIs)

**Fig. 1:**
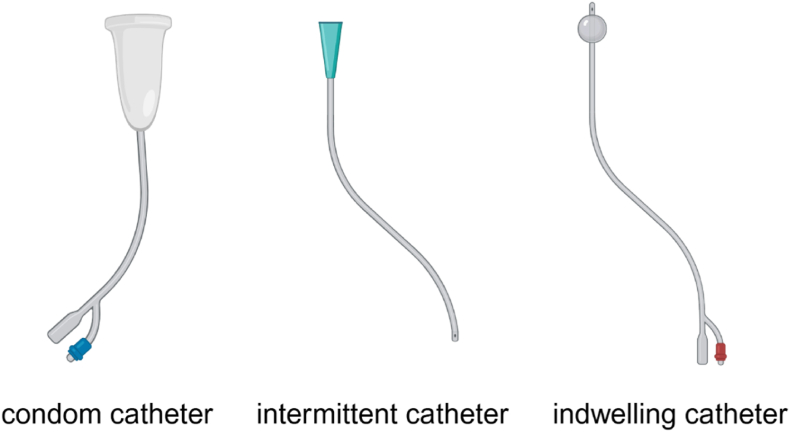
Different types of urinary catheters. Figure was created with BioRender.com.

The application of medical devices, ranging from simple wound care products to complex technologies such as implants, imaging systems and surgical robots, has significantly improved the quality of healthcare by providing more effective diagnosis and treatment [1]. Urinary catheters are one of the most common medical devices, which are deployed to manage bladder dysfunction (urinary retention or urinary incontinence) for those who have undergone surgery or have mobility issues, and about 15 %–25 % of individuals require urinary catheters during hospitalization [2,3]. Variosus polymeric materials, such as polydimethylsiloxane (PDMS), latex (natural rubber), polyvinyl chloride (PVC), and polyurethane, have been used to manufacture urinary catheters [2,4]. Based on the purpose and duration of use, urinary catheters can be broadly classified into three main types: condom catheters, intermittent catheters and indwelling catheters, as shown in Fig. 1. Condom catheters, also known as external catheters, are non-invasive devices worn over the penis like a condom. They are used in male patients with urinary incontinence for about one week and should be changed daily [5,6]. Intermittent catheters are used to empty the bladder for people suffering from urinary retention. Intermittent catheterization is a procedure where a catheter is temporarily inserted into the bladder to drain urine and then immediately removed once the bladder is empty. It is typically performed 4–6 times per day and can be done by patients themselves at home after proper training [7]. Indwelling urinary catheters (Foley catheters), the most widely used type of urinary catheter, were introduced by a urologist named Frederic Foley in the mid-1930s [5]. A typical Foley catheter consists of a flexible tube with two channels, one for urine drainage and one for inflation and deflation of the balloon at the end of the catheter. The balloon is inflated with sterile water from a syringe after the catheter insertion to hold the catheter in place within the bladder, preventing it from sliding out [8]. Indwelling catheters can be used for both males and females for short-term (up to 7 days) and long-term (longer than 28 days) catheterization, depending on the clinical indication [8,9]. Short-term indwelling catheters are generally used for acute urinary retention management, urine measurements, bladder irrigations, post-surgical urinary diversion and drainage, and for patients receiving epidural anaesthesia [10]. Long-term catheterization are typically used to treat chronic urinary retention for people with neurological disorders such as spinal cord injury, multiple sclerosis, or stroke [5,11].

According to a 2024 report by Global Insight Services, the global urinary catheter market value is projected to grow from $3.9 billion in 2024 to $7.4 billion by 2034, while sale units are estimated to increase from 620 million in 2024 to 900 million by 2028. Among the various types of catheters, indwelling catheters lead the market with a 45 % share [12]. Unfortunately, the use of indwelling urinary catheters often results in catheter-associated urinary tract infections (CAUTIs). Urinary tract infection (UTI) is one of the most prevalent healthcare-associated infections (HAI), accounting for nearly 40 % of all HAIs [13]. Approximately 70–80 % of UTIs are attributed to the use of indwelling urethral catheters [[14], [15], [16]]. If a CAUTI is left untreated, it may give rise to serious aftereffects like pyelonephritis, sepsis, bacteremia, and even death [17]. Thus, the healthcare costs related to CAUTI impose a considerable financial burden on the health system. According to reports in 2018 and 2019, the yearly economic burden related to CAUTIs was estimated to reach up to $1.7 billion in the United States and between £1.0–2.5 billion in the UK [18,19].

Despite the implementation of many protocols for the insertion and removal of urinary catheters across different hospital systems aimed at avoiding microbial contamination, such as handwashing, use of gloves, sterile barrier (*e.g.* sterile catheter kit, mask, cap, large drape and gown), no-touch insertion techniques and training, the risk of subsequent infections persists [20,21]. For hospitalized patients undergoing indwelling catheterization, the risk of acquiring a CAUTI increases by 3–7 % per day. In short-term catheterization (up to 7 days), 10–50 % of patients develop bacteriuria (the presence of bacteria in the urine), while infection occurs in almost all patients in long-term catheterization (more than 28 days) [5,22]. On one hand, urethral catheterization provides an abiotic surface for planktonic bacteria to attach, colonize and eventually form biofilms with high antimicrobial resistance [23]. The vulnerability of the urinary tract to CAUTIs is because the presence of an indwelling catheter compromises the natural defense mechanisms of the bladder against infection. Under normal physiological conditions, the regular bladder filling and emptying cycles help flush out bacteria that might have entered the urethra or bladder. However, during indwelling catheterization, urine drains through the catheter instead of flushing the urethra, allowing bacteria to easily migrate from the contaminated skin insertion site into the bladder through the urethra [5,24]. On the other hand, Foley catheters have a design flaw that leads to the inevitable development of bacteriuria during long-term use. Specifically, the drainage holes are typically located away from the bladder base due to the inflation balloon, creating a urine reservoir that can serve as a perfect environment for bacterial growth [25]. While some attempts have been made to design new indwelling catheters, structural modifications alone remain insufficient to prevent biofilm formation on catheter surfaces during long-term use [[26], [27], [28]]. Therefore, most current research focuses on developing antimicrobial materials and coatings to address CAUTIs.

### 1.2 Biofilm formation and catheter encrustation

Biofilm formation and catheter encrustation are the two main problems that make it challenging to treat CAUTIs. Understanding the underlying mechanisms of these processes is essential for developing effective strategies to manage CAUTIs.

#### 1.2.1 Biofilm formation

Bacterial biofilms are known as complex structured communities where bacterial cells are embedded in a self-produced sticky matrix of extracellular polymeric substances (EPS) [29]. According to studies, the EPS accounts for 90 % biofilm mass and mainly consists of proteins, polysaccharides, lipids, fatty acids, and various nucleic acids [30,31]. Bacteria can exist in two distinct states: the free-living planktonic state and the sessile, surface-attached state. Most bacteria in nature live within biofilms due to the survival advantage over the planktonic growth mode [30]. The EPS surrounding the biofilms can act as a barrier to protect the bacterial cells from the host immune system and various environmental stresses such as antimicrobial agents, UV, pH, starvation, and dehydration [32,33]. It has been reported that bacteria in biofilms are 10–1000 times more tolerant to antibiotics in contrast to planktonic cells [34].

**Fig. 2:**
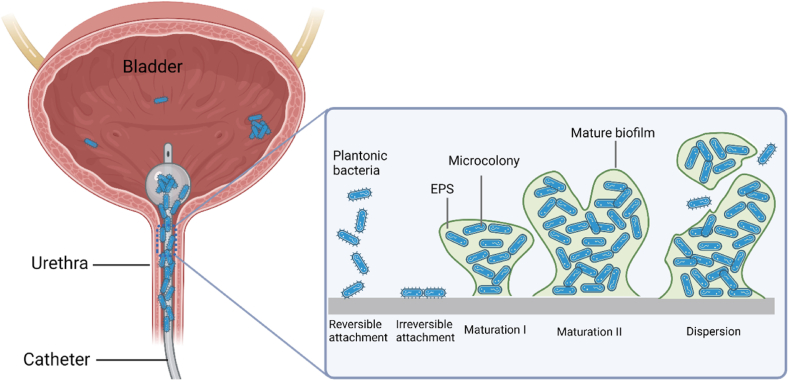
The process of biofilm formation on urinary catheter surfaces. Figure was created with BioRender.com.

Biofilm formation on a surface typically proceeds via several stages, including reversible attachment, irreversible attachment, maturation (maturation I and maturation II) and dispersion [35], as shown in Fig. 2. In the first stage, planktonic bacteria are transferred from bulk liquid to a surface either by physical forces or by bacterial appendages such as pili or flagella. Typical physical forces that are involved in bacterial adhesion include: hydrophobic, electrostatic (double layer) and Van der Waals interactions [30,36]. This stage is termed reversible attachment because of the weak interaction between bacteria and the surface, which can be easily affected by factors such as pH, temperature, pressure, and material composition [30]. When the attractive forces are greater than repulsive forces, the reversibly attached bacterial cells remain immobilized and become irreversibly adhered to the surface. Previous reports suggested that the physical appendages of bacteria, such as flagella, fimbriae and pili, overcome the physical repulsive forces between the electrical double layer of the cell and the surface (repulsive because of negative charge on both cells and the surface), thereby strengthening the surface-bacteria bond [36,37]. The irreversible attachment is followed by maturation, where the attached bacterial cells divide, leading to population growth, which activates the quorum-sensing (QS) system that allows microorganisms to communicate with each other [38]. The cell-to-cell communication relies on autoinducer signals, such as acylated homoserine lactone (AHL) [39]. Taking advantage of the QS system, bacteria control their behavior, like biofilm maturation, motility, and virulence factor expression. In addition, QS also promotes secretion of extracellular polysaccharides which contribute to the stabilization of biofilm structures [40]. In the biofilm maturation stage, the microcolony grows in size and increases the thickness to 100 μm, forming a three-dimensional macrocolony with diverse microbial communities in the biofilm [37]. In the final dispersion stage, the biofilm begins shedding and releasing daughter planktonic cells. Subsequently, the dispersed planktonic cells can translocate and attach to new sites and form new biofilms [41].

Adherence is a key step in initiating urinary tract infection (UTI). In uncomplicated UTI, pathogen may directly attach to the uroepithelium of the bladder, establishing a foothold for colonization [42]. In the case of indwelling catheters, infections typically start with bacterial adherence to the catheters, where microorganisms require fewer virulence factors to establish colonization and infection [43,44]. Once the catheter is inserted into the urinary system, a conditioning film forms quickly due to the deposition of urinary components such as electrolytes, proteins, and other organic constituents, which facilitates bacterial adhesion and the initiation of biofilm formation.

Microorganisms can enter the urinary tract via two major routes: extraluminal (along the outside surface of the catheter) and intraluminal (through internal channel of the catheter). It has been reported that most CAUTIs are caused by microorganisms that access through the extraluminal route (66 %) in both men and women, whereas 34 % of CAUTIs are intraluminally acquired [45]. Endogenous organisms that originate from the patient’s own gastrointestinal tract could be picked up by the catheter tip in distal urethra and pushed into the bladder during insertion of the catheter, or migrate along the catheter-urethral interface to the bladder after catheter insertion. These two possible ways constitute an extraluminal route of infection. Causative organisms of CAUTIs can also originate from exogenous sources, such as the hands of healthcare workers, and the skin of patients, as well as contaminated equipment [46]. These exogenous uropathogenic microbes may contaminate the collecting bag or drainage tube and then move into the bladder intraluminally because of manipulation of the closed drainage system, such as emptying the collecting bag or taking urine specimens [44,46].

The most prevalent bacteria associated with CAUTIs include *Escherichia coli (E. coli), Staphylococcus epidermidis (S. epidermidis), Proteus mirabilis (P. mirabilis), Pseudomonas aeruginosa (P. aeruginosa), Klebsiella pneumoniae (K. pneumoniae), Enterococcus faecalis (E. Faecalis), Staphylococcus aureus (S. aureus), coagulase negative Staphylococci and Morganella morganii (M. morganii)*. CAUTIs can also be caused by fungi like Candida species [42,47]. *E. coli* is known as the most common causative pathogenic organism in both complicated and uncomplicated urinary tract infections in humans, accounting for approximately 65–75 % of total cases [38].

Notably, some bacteria associated with CAUTIs are able to form crystalline biofilms, resulting in catheter encrustation and blockage, which may cause complications during long-term indwelling catheterization.

#### 1.2.2 Catheter encrustation and blockage

For long‐term urethral catheterization, the prevention of catheter encrustation and blockage due to the formation of crystalline biofilms is an important consideration. It is estimated that about 50 % of patients with long-term indwelling catheters will experience catheter encrustation and blockage [48]. The formation of crystalline biofilms results from infections by urease-producing bacteria such as *P. mirabilis*, *P. vulgaris, Providencia rettgeri.* [49].

**Fig. 3:**
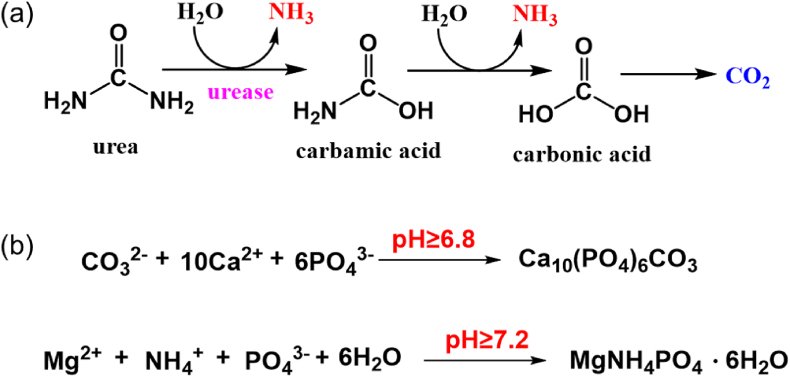
(a) The mechanism of urease-catalyzed hydrolysis reaction of urea [57] and (b) Formation of carbonate apatite and struvite [58].

Bacterial urease can decompose urea in the urine into ammonia and carbon dioxide, as illustrated in Fig. 3a. The resulting ammonia serves as a rich nitrogen source for the bacteria and elevates urine pH, consequently promoting the crystallization of magnesium ammonium phosphate (struvite) and calcium phosphate (carbonate apatite) [50,51]. Additionally, the released ammonia can break down the glycosaminoglycans layer which acts as a protective barrier of urothelial cells against bacterial infection, inducing damage and inflammation of urothelium [52]. The formation of carbonate apatite and struvite is shown in Fig. 3b. The elevated pH of the urine causes supersaturation of magnesium and calcium ions. When the urinary pH exceeds 6.8, carbonate apatite (Ca_10_(PO_4_)_6_CO_3_) precipitates as a minor component of the infectious urinary stone, whereas the main crystalline component, struvite (MgNH_4_PO_4_•6H_2_O), starts to crystallize when the pH of the urine rises above 7.2 [53]. Struvite crystallization exhibits first-order reaction kinetics at pH 8.0, 8.5, and 9.0. As pH rises from 8.4 to 9.0, the crystal growth rate significantly accelerates. Moreover, higher pH values also contribute to an increase in crystal density of struvite, with each pH unit elevation enhancing crystal aggregation by approximately 27.5–38 % [54]. As crystals become trapped within the developing biofilm, their growth is stabilized and enhanced by the biofilm matrix, causing gradual biofilm mineralization and ultimately leading to catheter blockage [55]. This in turn results in either urine leakage causing incontinence or urinary retention that could lead to painful bladder distention [24]. If the blocked catheters are not removed in time, severe consequences such as pyelonephritis, septicemia and endotoxic shock may occur due to the reflux of infected urine to kidneys [24,48]. In addition, the encrustation makes the removal of the catheter difficult as the deflation of the balloon is impaired and the rough crystalline deposits can cause great pain for patients and damage urethral mucosa during the removal process [56].

Among all the urease-producing organisms related to CAUTIs*, P. mirabilis* plays a predominant role in catheter encrustation, which has been isolated from ∼40 % of biofilms on catheters recovered from patients who experience long-term catheterization [49]. Moreover, the urease generated by *P. mirabilis* is highly active, which is capable to hydrolyze urea six to ten times faster than those generated by other species [59]. A previous study [60] demonstrated that in a laboratory model, *P. mirabilis* can elevate the pH of urine to above 8.3 and cause catheter blockage in about 20 h. In addition to the urease-producing ability, another signature feature of *P. mirabilis* is swarming mobility, a flagellum-dependent movement across a surface that enables the bacteria to rapidly migrate along catheters and enter the bladder, initiating infections [55,61].

Rough irregular surfaces also contribute to crystalline biofilm formation. It has been confirmed that irregularities around the eyeholes and striations on catheter surfaces derived from the manufacturing technique promote bacterial adhesion to the catheter [24,48]. Laboratory model experiments [62] used to investigate crystalline biofilm formation of *P. mirabilis* have demonstrated that initial bacterial adhesion usually occurs to the rough surface around the drainage eyeholes. Microcolonies establish within surface depressions and subsequently spread to the cover the entire rim of catheter eyelets. Crystals then appear in the biofilm, followed by spread of extensive crystalline biofilm along the catheter surfaces (either inner or outer surfaces) [56]. The encrustation can finally extend along the entire length of the catheter, but it develops most extensively below the eyeholes and usually obstructs the lumen at this site.

Crystalline biofilms also provide specific advantages for the bacteria. CAUTIs can often persist even after catheter removal, which may be due to the formation of crystalline biofilms [63]. When an encrusted catheter is removed, crystals can break off, and the crystal fragments containing viable bacteria again act as nuclei on which minerals grow, ultimately forming bladder stones. These bladder stones serve as pathogen reservoirs, reinfecting the bladder and promoting crystalline biofilm formation on a new catheter, thus perpetuating the cycle of infection [63,64].

### 1.3 Antibacterial coatings for urinary catheters

Considerable clinical efforts have been made to prevent or delay CAUTIs, such as systematic catheter replacement. However, replacing catheters frequently not only causes patient discomfort but also increases treatment costs. Moreover, bacteria colonization on indwelling urinary catheters may recur during long-term catheterization (occur at least twice a year) [65]. Therefore, many efforts have been devoted to developing antibacterial coatings for urinary catheters. Generally, there are two main types of antibacterial surfaces: bacteria-repelling (antifouling) and bacteria-killing (bactericidal) [[66], [67], [68]]. Antifouling surfaces can be constructed by functionalizing the surface with hydrophilic and/or zwitterionic polymers or by creating surface micro-topographies [[69], [70], [71], [72]]. Hydrophilic polymers such as poly(ethylene glycol) (PEG) interact with surrounding water molecules via hydrogen bonds and form a tightly bound water layer serving as a physical barrier to resist protein adsorption and bacterial adhesion [73]. Zwitterionic polymers such as polyphosphorylcholine, polysulfobetaine, and polycarboxybetaine have been widely studied for antifouling applications. The antifouling performance of polyzwitterionic coatings is also closely related to the formation of a hydration layer [74]. However, polyzwitterionic coatings exhibit greater hydrophilicity than PEG coatings, attributed to their enhanced interaction with water molecules through ionic solvation rather than hydrogen bonding, leading to excellent fouling performance [75]. Creating surface micro-topographies is another effective antifouling strategy. A lot of natural surfaces with complex topographies, such as lotus leaves, skins of marine animals like sharks and pilot whales, and the spiky surface of the sea urchin, have been confirmed to be effective in reducing bacterial adhesion [71,73,76]. The antifouling mechanisms of micro- and nanostructures are considered to be related to superhydrophobicity, capillary forces, attachment point minimization, and drag reduction [73]. Although antifouling surfaces are usually biocompatible and effective in inhibiting the adhesion of bacteria at the initial stage, they are unavoidably contaminated during long-term use because of the lack of bactericidal capacity, ultimately losing their efficacy in preventing bacterial growth and biofilm formation [66].

Bactericidal surfaces can be divided into two categories based on different mechanisms: release-killing and contact-killing [68,[77], [78], [79]]. At present, antibacterial urinary catheters used in clinical practice are typically produced using antibiotics- and silver-releasing materials [80]. Although both antibiotics and silver have been proven to be effective bactericides, the gradual decrease of the released agents will eventually lead to a loss of antibacterial efficacy, limiting their use in long-term indwelling catheters [80,81]. Previous research has demonstrated that silver-coated can only temporarily delay the early onset of CAUTIs, as evidenced by the increase in bacteriuria after more than one week of catheterization [82]. Desai et al. [83] found that commercial nitrofurazone-impregnated urinary catheters can only inhibit bacterial attachment for up to 5 days. In addition to the restriction on long-term use, the in *vivo* accumulation of silver ions would lead to potential cytotoxicity, while excessive use of antibiotics may initiate the development of drug-resistant bacteria and contribute to environmental pollution [[84], [85], [86]]. By comparison, contact-killing materials constructed via immobilization of bactericides like quaternary ammonium compounds and antimicrobial peptides show a longer antibacterial bioactivity, but this “killing” approach also suffers from several drawbacks, such as potential toxicity to mammalian cells and hemolysis (destruction of red blood cells) [[87], [88], [89]]. Another non-negligible problem related to bactericidal surfaces and especially to contact-killing surfaces is the accumulation of dead bacteria and debris, which not only hinders the contact between the bactericidal components and bacteria thus decreasing the bactericidal activity, but also acts as a conditioning film to supply nutrients for subsequent bacterial adhesion, resulting in immune response and inflammation [90,91].

Considering the limits of single-function antibacterial surfaces, the combination of antifouling and bactericidal strategies has been extensively explored to enhance the overall antibacterial efficacy. However, a straightforward integration of dual functionalities often leads to mutual interference, causing a decrease in the effectiveness of each individual mechanism [92]. Therefore, there is growing interest in developing smart coatings with on-demand bactericidal properties to achieve better antibacterial performance and biocompatibility while minimizing the risk of bacterial resistance.

### 1.4 Smart stimuli-responsive antibacterial coatings

Smart coatings are able to sense and rapidly respond to environmental stimuli, thus, they are also known as stimuli-responsive coatings. Depending on the sources of the stimuli, smart coatings are generally classified into physical and biological stimuli-responsive coatings. In the case of physically responsive coatings, the antibacterial ability can be activated by external stimuli such as light [[93], [94], [95], [96]], temperature [97,98], and electricity [99,100]. For example, Zhang et al. [101] constructed an antibacterial coating on polyurethane (PU) hernia meshes by using two natural polymers, gelatin (G) and hyaluronic acid (HA) and a synthetic boron dipyrromethene-based photosensitizer (BDP-6), PU-GHB in short. The prepared PU-GHB was able to kill bacteria quickly and efficiently upon the 808 nm near-infrared (NIR) irradiation because the BDP-6 molecules can efficiently convert NIR light into hyperthermia and reactive oxygen species (ROS) which in turn achieve a synergistic antibacterial effect by both disrupting the cell membrane and the metabolism of bacteria. Although external stimuli can be easily controlled, external stimuli-responsive systems always require the diagnosis of infections before the application of a stimulus, which may cause a lag effect and make it more difficult to cure infections. In addition, it is sometimes challenging to detect an infection, particularly with implanted devices [102,103].

By contrast, biological stimuli-responsive systems that rely on the presence of bacteria and their metabolites for activation are considered smarter and more promising. Therefore, many researchers also make great efforts to develop smart coatings that can respond to internal stimuli (*e.g.,* pH, enzymes, and toxin) in the specific microenvironment during infection. This class of coatings is also referred to as bacteria-responsive or infection-responsive coatings. Taking advantage of the difference between normal tissue and an infectious microenvironment, many antibacterial coatings have been developed. For example, bacteria-infected tissues often feature an acidic biofilm microenvironment due to the accumulation of organic acid, such as lactic and acetic acid, produced during bacterial metabolism [104]. Based on this characteristic, Shen’s group [105] designed and constructed a pH-responsive antibacterial coating on Ti-based implants. The antibacterial copolymer consisted of cationic quaternary ammonium salts (QAs) and carboxyl groups. Under normal physiological conditions, the negatively charged carboxyl groups shielded the positively charged QAs in the coating and avoided cytotoxicity. The cationic QAs were exposed and the bactericidal activity was initiated when an infection occurred because the carboxyl groups were protonated as the pH dropped.

There are already some excellent reviews about stimuli-responsive antibacterial materials [[106], [107], [108], [109]]. Therefore, this review focuses specifically on smart bacteria-responsive coatings designed for urinary catheters to combat CAUTIs.

## 2 Design of bacteria-responsive coating for urinary catheters

The accumulation of bacterial metabolites during bacterial colonization and propagation leads to a special microenvironment, markedly different from that of normal tissues. Consequently, these bacterial metabolites, such as enzymes and toxins, or the pH changes they induce, can serve as internal stimuli for designing responsive coatings to detect and/or treat infections. The following sections present pH- and bacterial metabolite-responsive coatings designed to combat CAUTIs (as summarized in Table 1).

**Table 1:** Summary of bacteria-responsive strategies used to combat CAUTIs presented in this paper.

Stimuli	Materials	Bacteria	Results	Ref.	Patent number
**pH**	cellulose acetate with bromothymol blue	*P. mirabilis*	gave warning signal on average 43 h before the catheters were blocked in *vitro* and 12 days *in vivo.*	[110,111]	US8062234 B2
**pH**	silicone with bromothymol blue and silica filler	*P. mirabilis,* *P. vulgaris,* *K. pneumoniae, P. rettgeri,* *M. morganii,* *E. coli,* *E. cloacae,* *P. stuartii,* *E. faecalis,* *P. aeruginosa, S. aureus*	detected the blockage of catheter on average 19 h in advance *in vitro* and ≥19 days *in vivo*.	[112,113]	EP1761162 B1
**pH**	PVA containing fluorescent dye sealed by Eudragit S100	*P. mirabilis*	provided a warning about 14.5 h before the catheter blockage *in vitro.*	[114]	EP3746013 A1; US20210030581 A1
**pH**	Eu(III)-based PHEMA hydrogel	biological experiments were not studied	demonstrated a fast and visible color change in response to urea hydrolysis	[115]	Not available
**pH**	PVA with bacteriophage sealed by Eudragit S100	*P. mirabilis,* *E. coli*	the *in vitro* blockage time of the coated catheters was doubled to 26 h, compared to 13 h for the uncoated catheters.	[116]	EP3746013 A1; US20210030581A1
**pH**	CS/CLG_NPs coating	*S. aureus,* *E. coli*	inhibit biofilm formation for at least one week *in vitro.*	[117]	Not available
**pH**	PHEMA-co-PMMA hydrogel loaded with nalidixic acid	*P. mirabilis*	good antibacterial activity toward *P. mirabilis*; faster drug release at infected urinary pH than at normal urinary pH, followed by a zero-order drug release over weeks.	[118]	Not available
**pH**	hydrogel of 2-HEMA and vinyl-functionalized nalidixic acid derivatives	*P. mirabilis*, *S. aureus*	the drug release rate was 20-fold faster at pH 10 than that at pH 7; ∼80 % reduction in adherence of *P. mirabilis* and *S. aureus* at pH 7 after 24 h; 95.6 % reduction in the adherence of *S. aureus* at pH 10 after 24 h.	[119]	Not available
**pH**	copolymers of 2-HEMA and derivatives Triton X-100	*P. mirabilis*, *S. aureus*	reduced the adherence of *P. mirabilis* and *S. aureus* by 85.6 % and 100 %, respectively, after 24 h incubation in infected artificial urine.	[120]	Not available
**pH**	hydrogel coating of PSBMA and TA loaded with antibacterial agents	*P. mirabilis*	excellent bactericidal activity toward *P. mirabilis*; remained encrustation-free surface for up to 7 days.	[121]	CN116102761A
**pH**	PAA, Eudragit S100, chitosan encapsulated polydiacetylene and ciprofloxacin	*P. mirabilis, S. aureus*	the blockage time of the coated catheter was longer than 30 h *in vitro,* nearly double that of the untreated catheter; reduced the time interval between signaling and blockage.	[122]	Not available
**pH**	PVA containing fluorescent dye and therapeutic agent sealed by Eudragit S100	*P. mirabilis*	provided an average of *ca.*79 h advanced warning of blockage, and the blockage time of the coated catheter reached up to 52.5 h.	[123]	Not available
**urease**	SA-PU/PVP	*P. mirabilis*	demonstrated almost no crystal deposition and lumens remained unobstructed in a porcine model for seven weeks.	[124]	Not available
**urease**	PU@pMDU	*K. pneumoniae,* *E. aerogenes*	achieved a bacterial inhibition rate of 75.3 ± 6.76 %; almost no bacteria were detected after 7 days of implantation in rat bladders.	[125]	Not available
**alkaline phosphatase**	coating of zwitterionic benzophenone-derived antibacterial agent	*S. aureus,* *E. coli*	showed good antifouling function at the first 2 h of incubation, and then switched to bactericidal function due to phosphatase response; both *in vitro* and *in vivo* models demonstrated the good bactericidal activity of the coating.	[126]	CN113801508B
**protease**	CHNS-EU&CP@β-CN	PAO1, MRSA	the coated catheters remained clean for 30 days *in vitro*, while uncoated catheters were blocked in 5 days.	[127]	Not available
**pyocyanin**	(HA/ACNSs)_10_	*P. aeruginosa*	the coated catheter remained free of biofilm after incubation for 7 days *in vitro*	[128]	Not available

### 2.1 pH-responsive coatings

An acidic microenvironment is a typical characteristic for a wide range of bacterial infections and most of the research has focused on the development of acid-responsive antibacterial coatings [[129], [130], [131]]. However, CAUTI is different from the majority of bacterial infections because it is always accompanied by an elevation in the pH of urine and biofilm due to the generation of ammonia from urea caused by urease-producing pathogens [60]. Using the rise in urinary pH as a trigger, a lot of infection-responsive coatings for urinary catheters have been developed, mainly including sensor coatings for CAUTI detection, drug-releasing antibacterial coatings and theranostic coatings (a combination of sensor and antibacterial coatings). The construction of pH-responsive coatings on urinary catheters entails the employment of pH-sensitive compounds such as Eudragit S100 (a copolymer of methacrylic acid and methyl methacrylate), polymers with ester groups and tannic acid (TA) [114,121,122]. The design of these pH-responsive systems are mainly based on the following mechanisms: 1) introduction of polymers protonatable groups, or 2) incorporation of polymers with alkaline-labile bonds and 3) using the disruption of the non-covalent interactions under alkalized conditions. For example, phenol groups in TA can be oxidized to quinones in basic conditions. These quinones have a lower ability to bind metal ions, leading to disassembly of the TA-metal network [121,132].

#### 2.1.1 Sensors for CAUTI detection

Great efforts have been made over the past decades to develop antibacterial coatings (*e.g.,* silver alloys and antibiotics) or antiadhesive coatings (*e.g.,* hydrophobic polytetrafluoroethylene and hydrophilic hydrogels) to prevent CAUTIs; however, all catheters currently available show limited effectiveness for long-term catheterization [133,134]. Silver-coated and antibiotic-coated catheters are the two main antibacterial urinary catheters currently on the market. Clinical studies have demonstrated that these catheters can inhibit infections for seven days at most, but fail to prevent biofilm encrustation for longer duration of urinary catheterization [[135], [136], [137]]. Due to the difficulty of completely preventing the development of catheter-associated bacteriuria (CAB), some researchers have turned to develop “early warning” systems that can quickly diagnose CAUTI so that patients can receive timely treatment.

In 2006, Stickler et al. [110,111] proposed the concept of using the increase of urinary pH as a stimulus to design a sensor for early warning of catheter encrustation and blockage. The sensor was prepared by impregnating cellulose acetate with bromothymol blue which can change color from yellow at pH 6 to blue at pH 8. However, the chemical synthesis method used to fabricate this sensor is thought to be not suitable for industrial-scale development. Therefore, in order to make the system easier to manufacture, the sensor was modified by incorporating bromothymol blue into silicone, along with a hydrophilic filler [112]. The developed sensor was placed in the junction between the catheter and drainage bag to monitor color change in response to different pH, which can predict the occurrence of catheter encrustation about 19 h in advance in *vitro.* Nevertheless, the time between sensor color change and blockage was much longer in human trials -- the warning from the modified sensor was ≥19 days in advance, raising doubts whether the sensor could be utilized as an indicator of imminent catheter blockage [113].

**Fig. 4:**
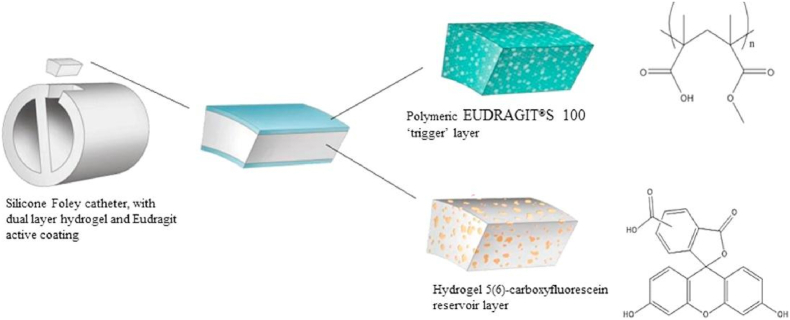
Schematic illustration of dual-layered polymeric architecture for pH-triggered release of 5(6)-carboxyfluorescein. Reproduced under terms of the CC-BY license [114]. Copyright 2016, The Authors, published by Elsevier.

In addition to the pH-sensitive dyes, another strategy for designing this kind of “early warning” sensor is using pH-sensitive polymers to control the release of dyes in response to pH elevation caused by *P. mirabilis*. Milo et al. [114] developed a coating sensor for urinary catheters based on Eudragit S100, a commercial copolymer of methacrylic acid and methyl methacrylate. The coating was composed of a two-layered hydrogel in which the lower hydrogel layer of poly(vinyl alcohol) (PVA) containing the dye carboxyfluorescein was sealed by the upper pH-responsive layer of Eudragit S100. The alkaline urine resulted in swelling of the outer Eudragit S100 layer, followed by release of fluorescent dye from the PVA hydrogel, giving a visual signal to warn the catheter blockage (Fig. 4). According to the results of an *in vitro* bladder model test, this coating sensor was capable of indicating infections ∼10–12 h prior to catheter blockage. This dual-layered hydrogel can also be fabricated as a lozenge sensor (a small tablet coated with the hydrogel) and directly placed in the drainage bag, providing a warning of catheter blockage approximately 14.5 h in advance [138]. Recently, this sensor system was further optimized by the same group for easy manufacture and commercialization, resulting in a system that can predict the occurrence of CAUTI 6.7 h before blockage [139].

**Fig. 5:**
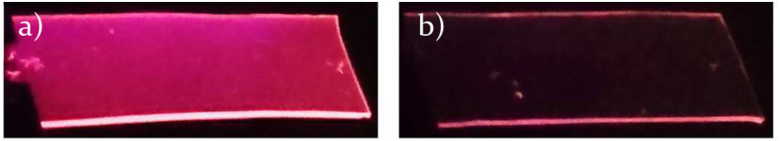
Photographs of the (a) “on” and (b) “off” state of the hydrogel, when irradiated at *λ*_max_ = 254 nm. The left image was acquired before the addition of urease and the right image 200 min after. Reproduced with permission [115]. Copyright 2017, American Chemical Society.

Surender et al. [115] reported a pH-responsive lanthanide-based luminescent probe that can monitor the pH change of urine related to urease-mediated hydrolysis of urea in both aqueous solution and water-permeable soft polymer materials. In detail, this sensor behaves as an “on–off” luminescent switching probe, where the luminescence can be quenched upon pH elevation to neutral/slightly alkaline due to the conversion of urea into ammonia and carbon dioxide. The Eu(III)-based probe can be encapsulated in hydrogels, serving as a potential sensor for the detection of CAUTIs. As shown in Fig. 5, the Eu(III)-based hydrogel sensor demonstrated a visible color change from bright red (on) to a faint pink (off) in response to the hydrolysis of the urea in solution after the addition of urease. Regretfully, this work focused on the proof-of-concept and did not explore the application of the developed sensor in urinary catheters.

#### 2.1.2 Drug-releasing antibacterial coatings

One of the major challenges in the treatment of bacterial infections is achieving efficient local delivery of antimicrobial agents to infection sites. In clinical practice, prolonged use of high-dose antibiotics is often necessary to achieve effective treatment of bacterial infections. This requirement is primarily attributed to the low bioavailability caused by the systemic distribution, with only a small fraction of antibiotics reaching the sites of infection [140]. However, the high-dose administration and overuse of antibiotics can lead to systemic side effects and accelerate the development of drug-resistant bacteria [141]. As alternative, drug delivery systems have been widely studied; however, traditional controlled drug delivery systems often rely on sustained passive delivery, which often limits the therapeutic efficacy due to premature drug leakage, slow and incomplete drug release, non-specific drug release [142]. Triggered release systems ensure drug release only in infected locations, thus enhancing their effective bioavailability and antibacterial properties while reducing damage to healthy tissues and the risk of developing antimicrobial resistance [143,144]. Hence, a wide range of stimuli-responsive, especially pH-responsive drug-releasing systems, have been developed to fight bacterial infections [[145], [146], [147]].

**Fig. 6:**
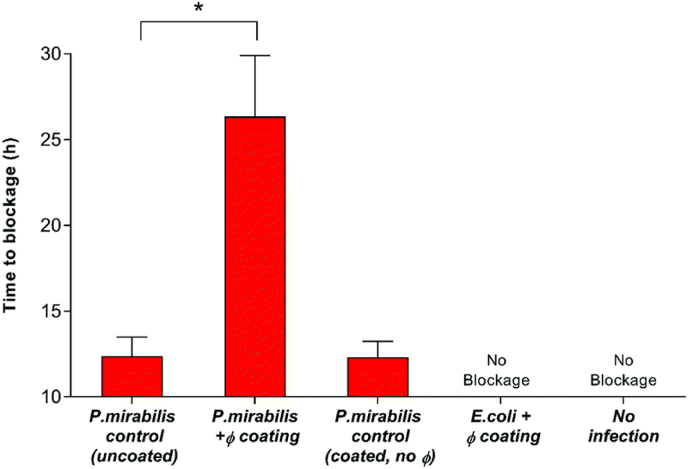
Impact of bacteriophage treatment on catheter blockage. *In vitro* models of the catheterised urinary tract replicating established *P. mirabilis* infection were used to evaluate the impact of triggered phage (denoted as *φ*) release on blockage and encrustation. The time at which the catheters became blocked and urine ceased to accumulate in the drainage bags was used as the experimental end point. Data represents the mean of 3 independent replicates. ∗*P* < 0.05. Error bars represent standard error of the mean (SEM). Reproduced under terms of the CC-BY license [116]. Copyright 2017, The Authors, published by Royal Society of Chemistry.

There are several approaches that have been explored to construct pH-responsive drug-releasing antibacterial coatings for urinary catheters. The first strategy involves the use of polymers with ionizable groups that can achieve conformational or/and solubility changes induced by the environmental pH variation. For example, Milo et al. [116] described a dual-layered pH-responsive coating system with bacteriophages as antibacterial agents for urinary catheters. This antibacterial coating comprised of a lower PVA layer with bacteriophage and an upper trigger layer of Eudragit S100. In the absence of urease-producing bacteria, the coating system remained stable. Once the urinary pH value increased to above 7 as a result of infections by urease-positive bacteria, the Eudragit S100 layer swelled due to the formation of the carboxylate anion. Consequently, the PVA reservoir layer was exposed to the urine media, leading to a burst release of bacteriophage from the coating. The study demonstrated that this coating can reduce *P. mirabilis* concentration by nearly 6 logs in a bladder model in 2 h upon activation by alkaline urine. As a result, the catheter blockage time of the bacteriophage-coated catheters was doubled to 26 h compared to 13 h for the uncoated catheters (Fig. 6), thereby extending the lifetime of catheters.

In another work, Puertas‐Segura et al. [117] developed a bio-based nanocomposite coating for silicone catheters by incorporating citronellal-loaded lauryl gallate nanoparticles (CLG_NPs) into a chitosan (CS) matrix using a sonochemical coating method. The coating demonstrated pH-dependent release of citronellal, which can be attributed to the pH sensitivity of CS. At pH6, higher protonation of amine groups of CS increased the interaction between CS and anionic CLG_NPs, limiting citronellal release. At pH values of 7 and 8, deprotonation of amine groups resulted in higher release of citronellal. The antibacterial performance of the coating evaluated by an *in vitro* bladder model showed the coated catheter was able to inhibit biofilm formation for at least one week.

Relying on the solubility variation of the drug itself at different pH to modulate drug releasing rate in response to the pH change is also a promising strategy to fabricate smart antibacterial coatings for urinary catheters. For example, Irwin et al. [118] developed a pH-responsive drug delivery system that can respond to the alkaline environment created at the onset of urinary catheter infections by loading nalidixic acid into polymer films of poly(2-hydroxyethylmethacrylate) copolymerized with hydrophobic methyl methacrylate (MMA). The nalidixic acid is an antimicrobial quinolone and its physicochemical properties are highly pH-dependent due to the presence of an ionizable carboxyl group in the molecular structure. In alkaline media, nalidixic acid showed a significantly improved solubility (10-fold increase) compared to its relatively low solubility at normal physiological urine pH. The study showed that the drug release rate of the designed system reached up to 50-fold faster at a typical infected urine pH than at normal urinary pH. Moreover, the pH-responsive drug release was followed by a prolonged period of zero-order release (release rates are constant) from the polymer films compared to the poly(2-hydroxyethyl methacrylate) (PHEMA) matrices due to the incorporation of hydrophobic PMMA. This sustained release profile suggests the possibility for this system to provide infection resistance for up to 4 weeks in the absence of intervention.

**Fig. 7:**
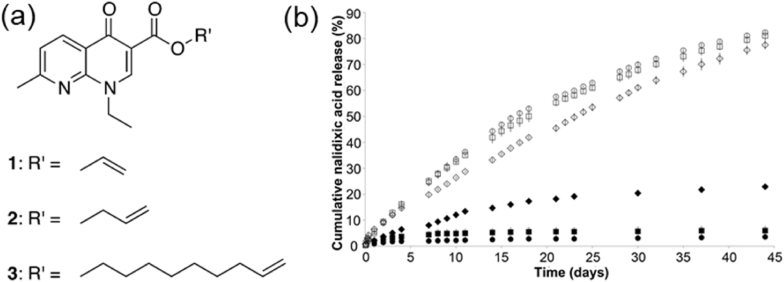
(a) Molecular structures of polymerizable nalidixic acid conjugates, (b) Effect of pH on the mean (±S.D.) release of nalidixic acid from copolymers 4–6 (prepared from the respective conjugates 1–3, 2-HEMA and EGDMA cross-linker) represented by circles, squares, and diamonds, respectively, at 37 °C with shaking. Sink conditions were maintained throughout the study. Open and closed symbols refer to release at pH 10 and 7, respectively. Reproduced with permission [119]. Copyright 2016, American Chemical Society.

Another important strategy to achieve pH-responsive release of antimicrobial agents is introducing alkaline-sensitive linkers like ester bonds. McCoy and coworkers [119] created a hydrogel drug delivery system that could act as alkaline-responsive coatings for antibacterial urinary catheters. In this work, a series of polymerizable drug conjugates were synthesized by covalently attaching the antibacterial agent, nalidixic acid, to alkenyl spacer moieties of different vinyl chain lengths via hydrolytically labile ester bonds (Fig. 7a), which were then copolymerized with 2-hydroxyethylmethacrylate (2-HEMA) and the cross-linker ethylene glycol-dimethacrylate (EGDMA) to form a hydrogel. An *in vitro* drug release kinetics study showed that the ester bond remained stable at normal physiological urine pH, with negligible release of nalidixic acid from the copolymer hydrogel. By contrast, the drug release rate significantly increased up to 20-fold faster at pH 10, representing infected urine pH. According to a previous study [118] from the same group, over 90 % nalidixic acid was released from physically loaded PHEMA hydrogel within 1 h at pH 7 and pH 9. However, the work here showed that ∼80 % of nalidixic acid was released from the hydrogel in 6 weeks at pH 10 (Fig. 7b), verifying the release mechanism as ester hydrolysis controlled. Additionally, the in *vitro* microbiological test showed that the designed system reduced adherence of *P. mirabilis* and *S. aureus* by ∼80 % after 24 h at pH 7 and achieved a 95.6 % reduction in the adherence of *S. aureus* at pH 10 after 24 h.

**Fig. 8:**
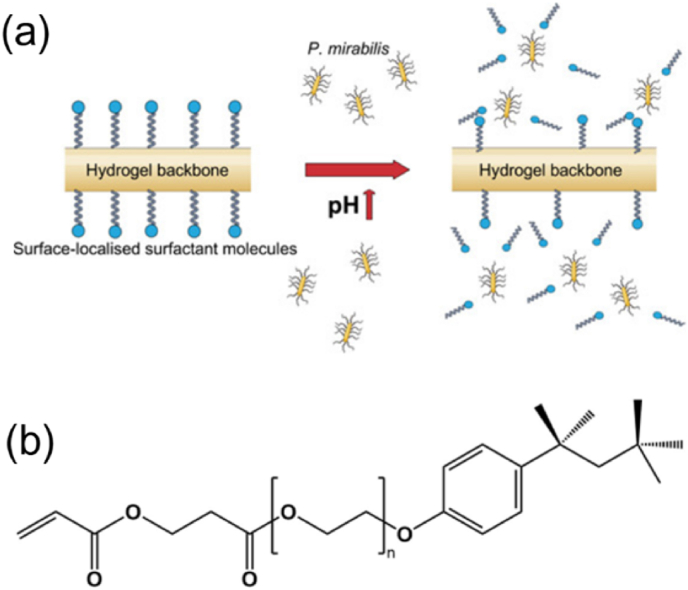
(a) Illustration of pH-responsive release of TX molecules under the alkaline conditions typical of catheter-associated urinary tract infections and (b) TX conjugate. Reproduced with permission [120]. Copyright 2021, American Chemical Society.

An infection-triggered, self-cleaning hydrogel system reported by Irwin et al. [120] also integrates the hydrolytically labile ester bond. Considering the risk of bacteria resistance using antibiotics, the authors chose the nonionic surfactant Triton X-100 (TX) as an active antimicrobial agent to develop a stimuli-responsive antifouling surface, which combined the passive fouling resistance exhibited by surfactant agents with active and responsive release of surfactant moieties, associated bacteria and particulate substance from the surface. The hydrogel was prepared by copolymerizing the dual-functionalized surfactant TX containing alkali-cleavable ester bonds and vinyl groups (Fig. 8) with 2-hydroxyethyl methacrylate (2-HEMA). The surfactant release from the copolymer hydrogel was pH dependent: at pH 7, the rate for 50 % release of the conjugated TX moiety from the hydrogel was about 5-fold lower than at pH 10. After 24 h of incubation in infected artificial urine, the copolymers displayed complete resistance to the adherence of *Staphylococcus aureus* and up to 85.6 % reduction in the adherence of *Proteus mirabilis*.

**Fig. 9:**
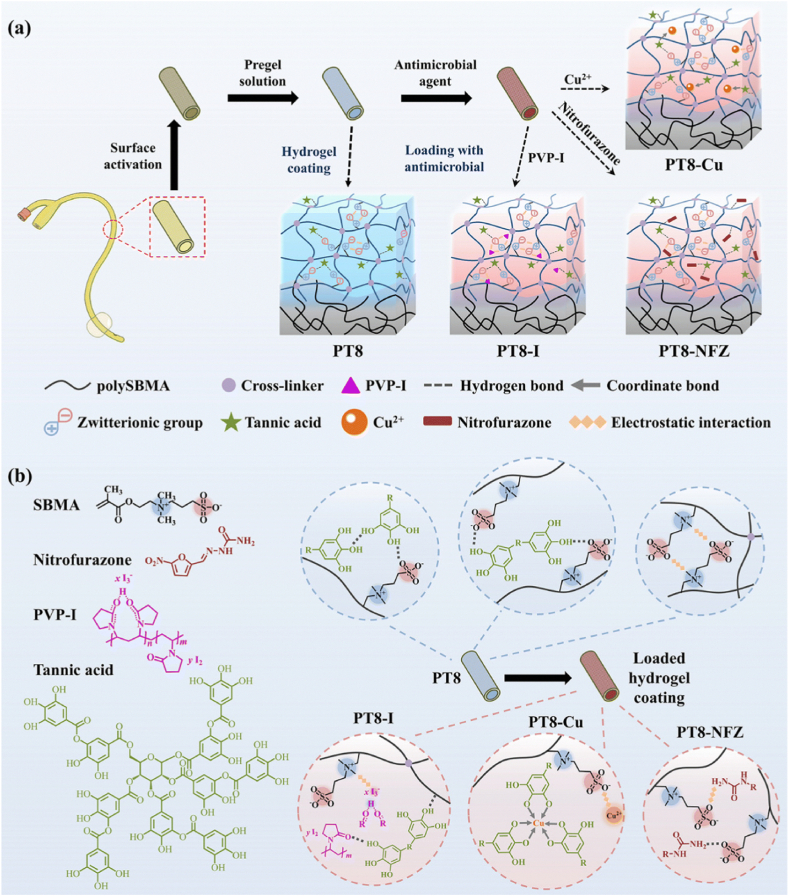
Schematic diagram illustrating a) the preparation process of a multifunctional hydrogel coating on a urinary catheter surface, and b) the structure of PT8 hydrogel coating and antimicrobial-loaded hydrogel coating (i.e. PT8-I, PT8-Cu, and PT8-NFZ). Reproduced under terms of the CC-BY license [121]. Copyright 2023, The Authors, published by Royal Society of Chemistry.

Disruption of non-covalent interactions between the antimicrobial agents and the drug-loaded coating under alkalized conditions has also been applied as a strategy to construct pH-responsive coatings for urinary catheter applications. Miao et al. [121] described a hydrogel coating of polysulfobetaine methacrylate (polySBMA) and tannic acid (TA) loaded with different types of antibacterial agents like poly(vinylpyrrolidone)-iodine (PVP-I), copper ions or nitrofurazone (the coatings were denoted as PT8-I, PT8-Cu and PT8-NFZ, respectively). The preparation process of the hydrogel coating is shown in Fig. 9a. Owing to multiple binding sites for non-covalent interactions, including coordinate bonding, electrostatic interactions and hydrogen bonding provided by the TA molecules and zwitterionic groups (Fig. 9b), the coating demonstrated a high loading efficiency with the antimicrobial agents. When the hydrogel coatings were immersed in buffer solution at pH 10 for 7 days, the released amounts of iodine, copper and nitrofurazone were up to 2.37 times, 53.8 times, and 112 times higher, respectively, as compared to 7 days immersion in a pH 7 solution. The pH-responsive release of the antimicrobials was due to the disruption of the non-covalent interactions between the antimicrobial agents and hydrogel coating under alkalized conditions. Meanwhile, the hydrogen bonding between TA and polySBMA was also destroyed at higher pH, which further accelerated the drug release from the hydrogel. The results indicated that the PT8-I coating showed the best antibacterial and anti-encrustation performance, which remained encrustation-free for up to 7 days according to both *in vitro* and *in vivo* studies.

#### 2.1.3 Theranostic coatings

The term “theranostics” refers to an integrated system with the ability to perform both diagnostic and therapeutic functions simultaneously or sequentially [148,149]. The concept has been widely used for the design of novel drug delivery systems in the field of cancer in order to achieve early detection of the disease and instantaneous treatment [149]. Since diseases like cancer are highly heterogeneous and all available treatments are effective for very limited patient subpopulations and at specific stages of disease development, theranostic approaches seek to provide more individualized and customized therapies, which are thought to be able to improve therapy’s precision and efficiency [150,151]. This concept has recently found application in the management of CAUTIs.

**Fig. 10:**
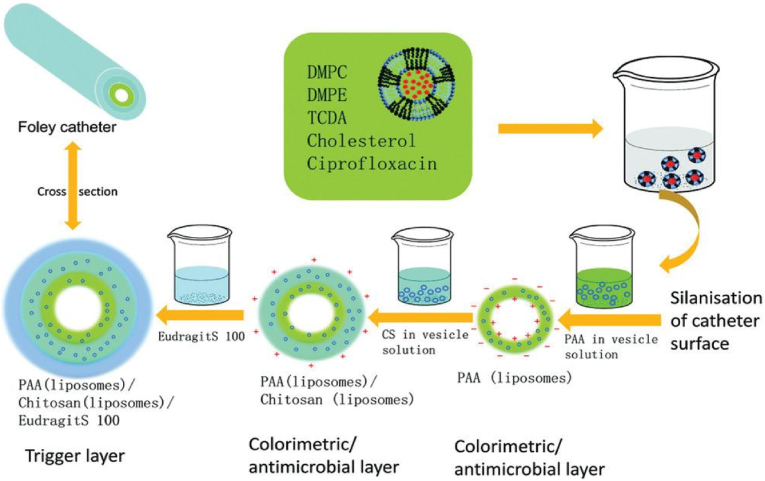
Schematic illustration of multilayer polymer on catheter surface by electrostatic self-assembly technique. PDA vesicles are embedded in a colorimetric layer and sealed by an outer layer of pH sensitivity. Reproduced with permission [122]. Copyright 2018, Wiley-VCH.

Zhou and co-workers [122] reported a theranostic multilayer coating that can provide early visual warning of catheter blockage and inhibit bacterial growth. By using the electrostatic self-assembly (ESA) approach, the coating was prepared with an inner layer of hydrogel PAA (poly (acrylic acid)), a middle layer of CS (chitosan) and a pH-responsive layer of Eudragit S100, as shown in Fig. 10. The PAA layer and CS layer were used to encapsulate polydiacetylene vesicles containing the antibiotic ciprofloxacin HCl, while the top Eudragit S100 layer was employed to seal the inner and middle layers to ensure no antibiotic delivery vesicles release from the coating in a normal urine environment. When urinary pH increased due to infections of urease-producing bacteria like *P. mirabilis* and *S. aureus*, the outer layer of Eudragit S100 dissolved, followed by the release of polydiacetylene (PDA) vesicles into the infected urine. PDA vesicles can change colour at physiological temperature, from blue in acidic media (pH < 7) to purple at pH 7–8.8 and red at pH > 8.8. Meanwhile, the release rate of the antimicrobial was about 2.5 times higher under alkaline conditions, compared to neutral and acidic conditions, thus slowing down bacterial growth. The coating demonstrated inhibition ability against both *P. mirabilis* and *S. aureus,* as evidenced by bacterial cell reduction percentages of 98.3 ± 0.05 % and 87.7 ± 0.02 %, respectively. As a result, *P. mirabilis* and *S. aureus* hardly blocked the catheters coated with the multilayer hydrogel containing ciprofloxacin HCl, with the average blockage time longer than 30 h in residual bladder models. By contrast, the coated catheters without antimicrobials were completely blocked after ∼15 h by *P. mirabilis* and ∼17 h by *S. aureus*, suggesting the potential of the proposed sensor coating loaded with antimicrobials in prolonging the life time of indwelling catheters.

Slate et al. [123] also designed a catheter coating that can simultaneously detect CAUTIs induced by urease-positive bacteria and actively reduce biofilm formation to prevent catheter blockage. The coating was developed based on the previous work of their group as described above [114], which consisted of an upper pH-sensitive Eudragit S100 layer and a lower PVA hydrogel layer incorporating self-quenching fluorescent dye 5(6)-carboxyfluorescein (CF). In this study, a therapeutic agent (either the urease inhibitor acetohydroxamic acid (AHA) or the antibiotic ciprofloxacin hydrochloride (CIP)) was also introduced into the PVA layer to treat infections immediately while impending catheter blockage was detected. An *in vitro* bladder model study demonstrated that the combination of CF dye with CIP significantly increased the average advanced warning time of blockage to ∼79 h and extended catheter lifespan by 3.40-fold, in comparison with the empty coatings (without CF dye and therapeutic agents), while the coating containing CF dye and AHA illustrated a limited increase of blockage time to ∼36 h. This study further confirmed the possibility of theranostic coatings as a promising strategy against the encrustation and blockage of urinary catheters.

### 2.2 Bacterial metabolite-responsive coatings

In addition to the pH variation, bacteria also produce a variety of substances during metabolism, such as enzymes (*e.g.* hyaluronidase, phosphatase, β-lactamase, urease, gelatinase, lipase and protease) and toxins [[152], [153], [154], [155], [156], [157], [158], [159], [160]], which are specific biomolecules in the microenvironments of infections and can be used as triggers to design intelligent antibacterial coatings. Although there is a wide range of research related to bacterial metabolite-responsive coatings, in the field of antibacterial urinary catheters, enzyme- and toxin-responsive coatings are still in their infancy and only very few examples used enzymes and/or toxins as stimuli.

#### 2.2.1 Enzyme-responsive coatings

**Fig. 11:**
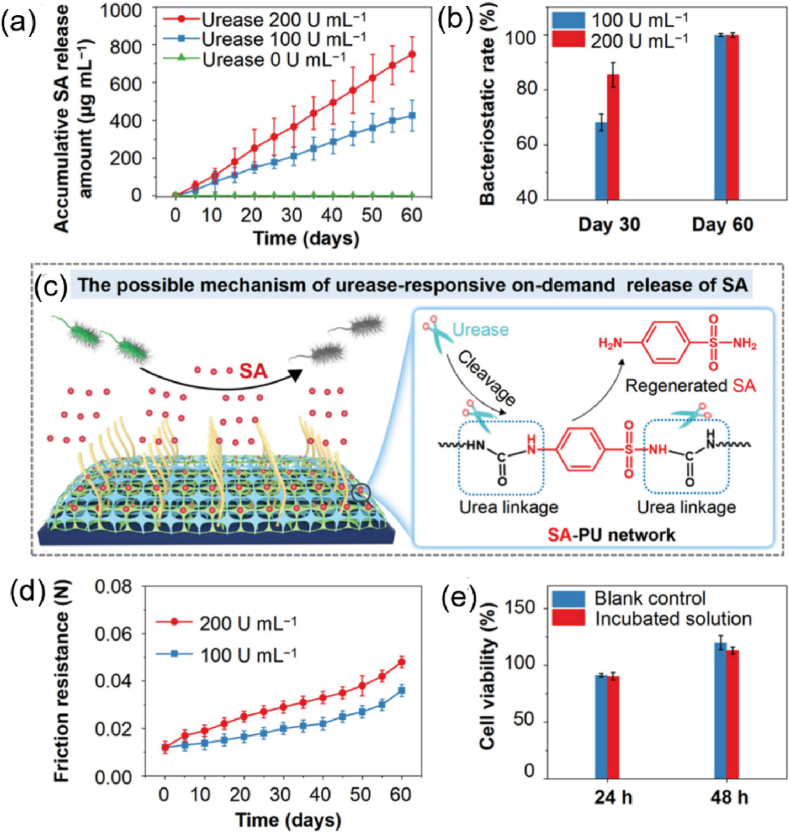
(a) Accumulated SA released from the SA-PU/PVP coating incubated in different concentrations of urease solution within 60 days. (b) Bacteriostatic efficiency of urease-incubated solutions at day 30 and day 60 against *P. mirabilis*. (c) Schematic illustration of the possible mechanism of urease-responsive on-demand release of the antibiotic SA from the SA-PU/PVP coating. (d) Changes in the friction resistance of the SA-PU/PVP coating incubated in different concentrations of urease solution within 60 days. (e) Cell viability of L929 cells cocultured with urease-incubated solutions at day 60 for 24 and 48 h. Reproduced with permission [124]. Copyright 2023, Wiley-VCH.

Urease is a crucial enzyme secreted by a variety of microorganisms associated with CAUTIs, such as *P. mirabilis, P. vulgaris and P. rettgeri*. The elevation of urine pH results from the hydrolysis of urea by urease, and a lot of research focuses on using pH instead of urease as a stimulus to develop smart materials for urinary catheters. Only recently, Li et al. [124] reported an urease-responsive antibacterial coating (SA-PU/PVP) by incorporating the antibiotic sulfanilamide conjugated polyurethane (SA-PU, linked via urea bonds) with urea linkage into a commercial polyvinylpyrrolidone (PVP)-based lubricant. In the absence of urease, almost no SA release from the SA-PU/PVP coating was detected during the 60 days of incubation. In the case of incubation with urease solutions, a continued release of SA was observed because of the cleavage of urea linkages, and the cumulative amount of released SA was proportional to the urease concentration (Fig. 11a). The bactericidal activity of SA was well preserved during the covalent conjugation and enzymatic cleavage processes, as evidenced by the high bacteriostatic efficiency of the regenerated SA catalyzed by urease (Fig. 11b). Meanwhile, good biocompatibility of the released SA was confirmed (Fig. 11e). In addition, the incorporation of hydrophilic PVP not only endowed the coating with good lubrication performance, but also reduced bacterial adhesion by more than 99.9 %, indicating excellent antifouling ability. Fig. 11d revealed that the lubricating activity of the coating remained high for 60 days even after the coating was incubated in urease solution, suggesting great stability of the coating. Notably, the SA-PU/PVP-coated ureteral stents demonstrated almost no crystal deposition and maintained unobstructed lumens in a porcine model over seven weeks. These results confirmed the potential of this coating strategy to prevent complications associated with urological medical devices.

**Fig. 12:**
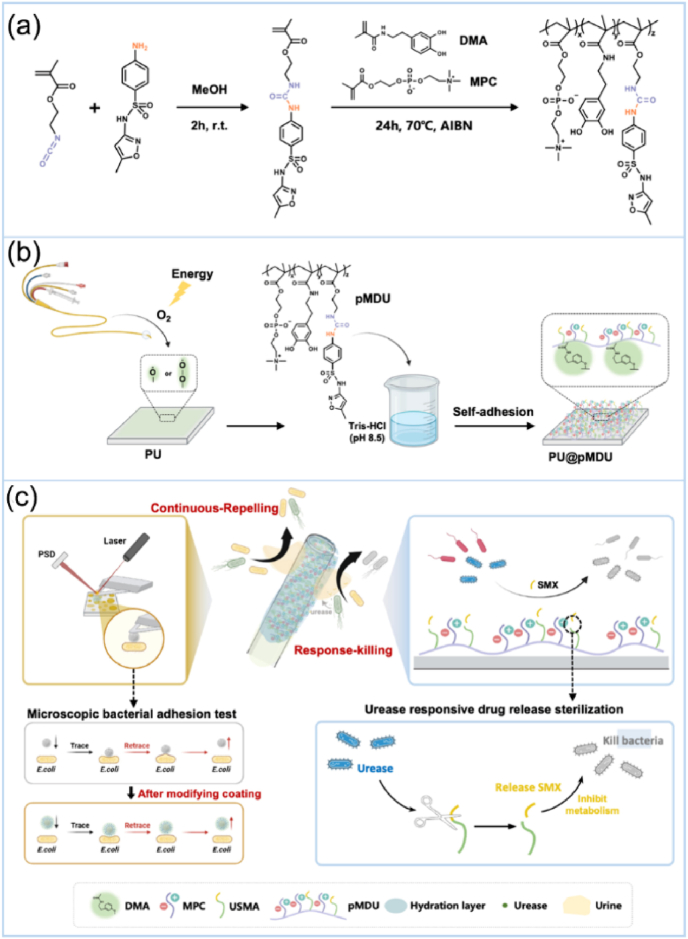
Schematic illustration showing the design mechanism of a urease-responsive lubricating antibacterial coating and its application in urinary tract implantable devices. (a) Chemical synthesis of the pMDU coating. (b) Preparation of the urease-responsive lubricating surface by dip coating. (c) Synergistic effect of the antibacterial and urease-responsive bactericidal abilities of the pMDU coating. Reproduced with permission [125]. Copyright 2025, American Chemical Society.

Jia et al. [125] developed a coating featuring antifouling and urease-responsive bactericidal functions for urinary tract implantable devices. The authors designed a monomer containing a urea group and the antibiotic functional group sulfamethoxazole (SMX), and then copolymerized it with other functional monomers to obtain pMDU (Fig. 12a). This polymer was then used to coat polyurethane (PU) substrates (Fig. 12b). The pMDU coating remains inactive under normal physiological conditions, but selectively releases SMX upon exposure to urease, enabling precise and on-demand drug delivery, as illustrated in Fig. 12c. The urease-responsive drug release study demonstrated that no SMX was released from the pMDU coating under urease-free conditions. However, in PBS solution containing 200 U/mL urease, SMX was almost completely released within 18 h, compared to 32 h with 100 U/mL urease*. In vitro* antibacterial tests revealed that the PU@pMDU can realize a bacterial inhibition rate of 75.3 ± 6.76 % compared to the uncoated PU substrate. To further validate the urease-triggered bactericidal activity of the coating, an *in vivo* antibacterial test was performed by implanting the PU and PU@pMDU samples, preincubated with urease-producing *E. aerogenes*, into rat bladders. After 7 days, the uncoated PU samples exhibited a total bacterial count of 1.13 × 10^6^ CFU, while bacterial colonization on PU@pMDU samples was nearly undetectable. In addition, scanning electron microscope (SEM) images revealed bacterial death on PU@pMDU samples. These results demonstrated the dual functionality of the pMDU coating in inhibiting bacterial adhesion and killing bacteria.

**Fig. 13:**
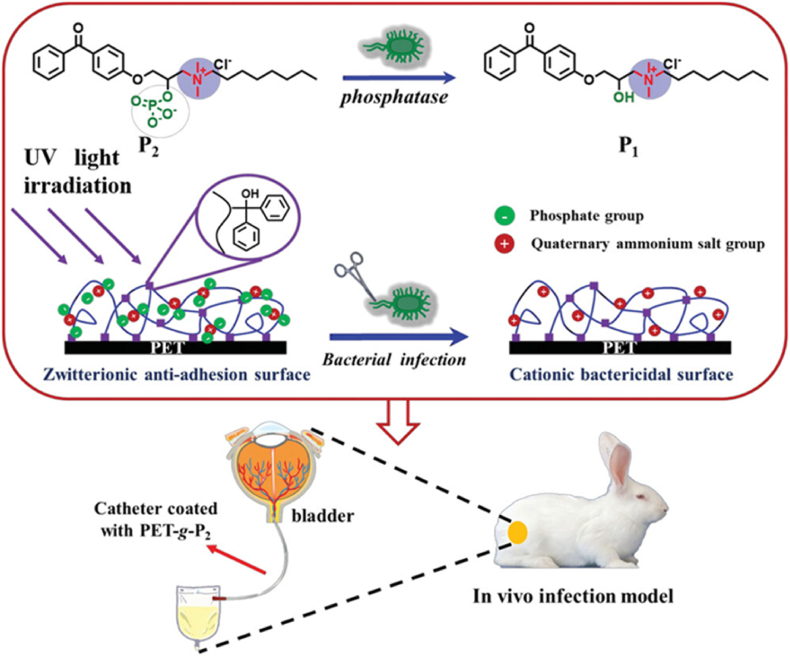
Schematic illustration showing the alkaline phosphatase-responsive surface biomaterials for urinary tract infection therapy *in vivo*. P1 was N-(3-(4-benzoylphenoxy)-2-hydroxypropyl)-N, N-dimethyloctan-1-aminium and P2 was N-(3-(4-benzoylphenoxy)-2-(phosphonooxy) propyl)-N, Ndimethyloctan-1-aminium. PET-g-P1 and PET-g-P2 are photoinitiated grafts of P1 and P2 onto the surface of polyethylene terephthalate (PET) material, respectively. Reproduced with permission [126]. Copyright 2023, Wiley-VCH.

Another essential bacterial enzyme, alkaline phosphatase, is primarily found in the periplasmic area of the bacteria surface wall and is related to the outer membrane [161]. Both gram-positive and gram-negative bacteria are capable of secreting alkaline phosphatases, which can catalyze the hydrolysis of phosphate esters into inorganic phosphate and alcohol in a nonspecific but highly efficient manner [162,163]. Therefore, significant interest has been attracted for designing phosphatase-responsive antibacterial systems [153,164,165]. Zhang et al. [126] described a phosphatase-responsive antibacterial surface for the application of urinary catheters. In their work, a synthetic zwitterionic benzophenone-derived antibacterial agent (P2) that is sensitive to alkaline phosphatase was covalently attached to the surface of common catheter materials, like polypropylene (PP) and polyethylene terephthalate (PET), to create the smart antibacterial surface by using a simple light-triggered technique. Due to the hydrophilic zwitterion structure of the antibacterial agent, the constructed intelligent surface exhibited excellent antifouling properties and biocompatibility. At infectious sites where bacteria secreted a large amount of alkaline phosphatase, the phosphate groups were removed, and the bactericidal ability of the surface was activated, as shown in Fig. 13. *S. aureus* and *E. coli* were used as two model bacteria to investigate the change of the surface antifouling performance of PP, PP-g-P1, and PP-g-P2 under continuous bacterial growth. The results showed that almost no bacteria adhered to PP-g-P2 in the first 2 h, whereas a certain amount of bacterial adhesion on PP and PP-g-P1 was observed. As time prolonged, the number of dead bacteria on PP-g-P2 increased along with the rising amount of bacterial adhesion, indicating that the PP-g-P2 surface experienced a phosphatase reaction under conditions of bacterial proliferation. In comparison, virtually no bacterial adhesion was found on the PP-g-P2 surface with alkaline phosphatase inhibitors in the solution after 24 h of incubation, while a considerable amount of bacteria remained adherent to the surfaces of PP and PP-g-P1. These results revealed that the transition of the PP-g-P2 surface from anti-adhesion to sterilization was achieved by alkaline phosphatase-triggered hydrolysis. Additionally, two models, including *in vivo* infected tissues and *in vitro* infected materials, were constructed to evaluate the antibacterial performance of the PET-g-P2 surface. Both of the two models demonstrated that the PET-g-P2 surface possessed bactericidal activity before and during implantation.

**Fig. 14:**
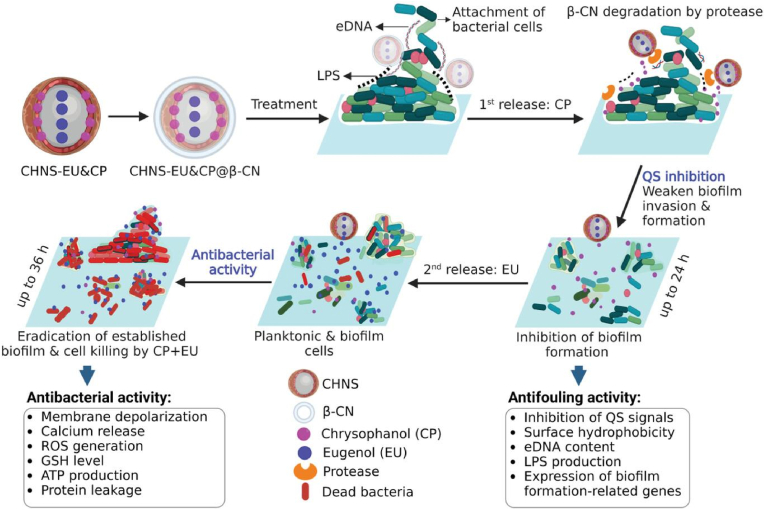
Schematic representation of the antifouling activity of a bacteria-responsive nanosystem (CHNS-EU&CP@β-CN), which effectively inhibits bacterial QS and potentiates the chemotherapy of the bactericide for eradicating established biofilms and killing bacterial cells through different mechanisms of action. Reproduced with permission [127]. Copyright 2023, Wiley-VCH.

Broad-spectrum bacterial pathogens also express proteases, which are a class of enzymes that can cleave peptide bonds [166]. Prateeksha and coworkers [127] designed a bacteria-responsive multidrug delivery nanosystem based on chitosan hollow nanospheres (CHNS). As shown in Fig. 14, first the bactericide eugenol (EU) was encapsulated followed by the QS inhibitor (QSI) agent chrysophanol (CP) into CHNS. Subsequently, the obtained CHNS-EU&CP were coated with beta-casein (β-CN) (denoted as CHNS-EU&CP@β-CN). In the microenvironment of bacterial infections, the β-CN layer was degraded by proteases, followed by the sequential release of CP and EP to inhibit biofilm formation. As a QSI agent, the CP can improve the sensitivity of bacteria to bactericide effectively by decreasing surface hydrophobicity, eDNA content and lipopolysaccharide production in biofilms, thereby enhancing the chemotherapeutic (medicines to kill fast-growing cells for treatment of cancer or infections) effect of EU. *Pseudomonas aeruginosa* (PAO1) and methicillin-resistant *Staphylococcus aureus* (MRSA) were used to evaluate the anti-QS and antibacterial properties of the nanosystem. Upon incubation for 36 h, CHNS-EU&CP@β-CN (50 μg mL^−1^) exhibited over 98 % bactericidal activity against both PAO1 and MRSA. By contrast, CHNS-EU@β-CN at the same concentration exhibited poor antibacterial effects on MRSA and PAO1 of 42.7 % and 37.6 %, respectively. In addition, the PDMS surface coated by CHNS-EU&CP@β-CN significantly suppressed the formation of bacterial biofilms, with 1.8 logs reduction in PAO1 biomass and 1.9 logs reduction in MRSA biomass, compared to uncoated surfaces. The nanosystems were further applied on commercial silicon catheters via an impregnation process to study the long-term antifouling activities of the coating. The results showed that the coated catheters remained clean for 30 days under artificial urine flow, whereas the untreated catheters were completely blocked within 5 days after inoculation with the PAO1 and MRSA cells.

#### 2.2.2 Toxin-responsive coatings

**Fig. 15:**
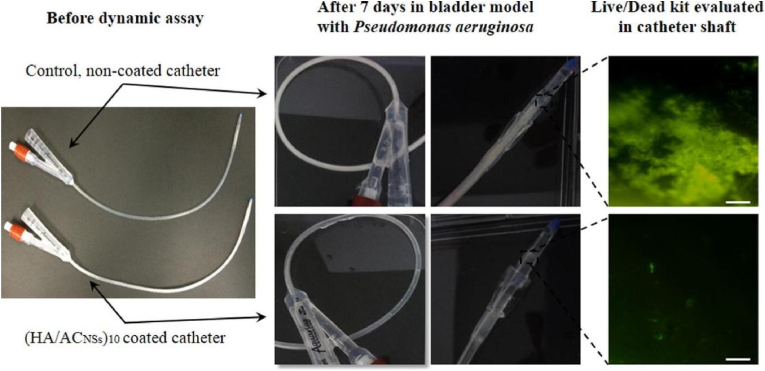
Photos and fluorescence microscopy images (20 × magnification) of the shaft sections of untreated Foley catheter (top row) and catheter coated with (HA/AC_NSs_)_10_ (bottom row) after a 7-day incubation in a dynamic bladder model system with *P. aeruginosa*. Scale bars 100 μm. Reproduced with permission [128]. Copyright 2016, Elsevier.

Toxins generated by bacterial pathogens during the infection process can damage host tissues, which allows the bacteria to penetrate deep into tissues [142]. Bacterial toxins have also been exploited as stimuli to develop antibacterial systems. For example, Francesko et al. [128] reported a layer-by-layer coating of antibacterial polycationic aminocellulose nanospheres (AC_NSs_) and hyaluronic acid (HA) polyanion on silicone urinary catheters in response to pyocyanin, a toxin produced by *Pseudomonas aeruginosa* (*P. aeruginosa*) which is a pathogen related to most clinical urinary tract infections. The auto-oxidation characteristics of pyocyanin allow it to break down glycosaminoglycans such as HA through a non-enzymatic mechanism. The coating was stable in the case without bacteria, while in the presence of *P. aeruginosa*, the HA was degraded gradually, thus releasing antimicrobial agents of AC_NSs_ and leading to a 70 % decrease in planktonic bacterial growth. Furthermore, an *in vitro* catheterized bladder model was used to test the dynamic antibiofilm properties of Foley catheters coated with (HA/AC_NSs_)_10_. As shown in Fig. 15, the formation of bacterial biofilms on the untreated catheter was clearly observed after 7-day incubation in the bladder model with *P. aeruginosa,* while the coated catheter remained free of biofilm.

## 3 Conclusions and future perspectives

CAUTIs are one of the most common hospital-acquired infections (HAI) due to the widespread use of urinary catheters in healthcare settings. The high risk of infection, as well as the relative clinical and economic costs of CAUTIs, motivated the development of antibacterial urinary catheters. However, all commercially available antibacterial urinary catheters (*e.g.,* silver- and antibiotic-releasing catheters) are not satisfactory for long-term indwelling catheterization. In addition to the problems of biofilm formation and encrustation, another challenge in managing CAUTIs is the emergence of antibiotic resistance in microbes and their decreased drug effectiveness over time. In this context, the design of smart stimuli-responsive antibacterial coatings can be an appealing alternative strategy against CAUTIs. In contrast to conventional antibacterial coatings, stimuli-responsive coatings offer significant advantages including timely detection of infections and on-demand activation of bactericidal functions, thereby preventing the development of bacterial drug resistance, inhibiting biofilm formation and reducing the cytotoxicity of antimicrobial agents.

In this review, we summarize the works of smart bacteria-responsive coatings for combating CAUTIs in recent years. The design of these types of coatings is based on the change of the microenvironments when a CAUTI occurs, such as elevated urine pH, overexpressed bacterial enzymes, and toxins. Among these internal stimuli-responsive coatings for urinary catheters, pH-responsive systems have been most extensively explored. However, bacterial metabolites-responsive coatings, particularly enzyme-responsive coatings are considered to be very promising in antibacterial application due to their specific and selective bactericidal effects. The advantages and disadvantages of different types of smart coatings are summarized in Table 2 [[167], [168], [169]]. Among the described systems, sensor coatings that provide visual warning signals of infection deserve special attention due to their importance in the early detection of CAUTIs and their potential for integration into theranostic platforms that enables prompt diagnosis and targeted antimicrobial treatment. Notably, many smart stimuli-responsive coatings integrate both bactericidal and antifouling components to provide synergistic antibacterial effects, thus achieving longer-lasting antibacterial effectiveness.

**Table 2:** Advantages and disadvantages of different types of bacteria-responsive coatings for combating CAUTIs.

Types of coatings	Advantages	Disadvantages
pH-responsive coatings	Simplicity of design: pH-responsive materials have been extensively studied and various pH-responsive materials are commercial	Low specificity to bacterial infection as urine pH may be affected by diet and other factors
Enzyme-responsive coatings	High specificity to bacteria and rapid response to bacterial enzyme	Spatial and temporal heterogeneity of enzymatic activity; complexity of synthesis and large-scale manufacturing
Toxin-responsive coatings	Very high specificity to pathogenic bacteria	Limited to toxin-producing bacteria

Despite recent advances in the field of smart antibacterial coatings for urinary catheters, most reported bacteria-responsive antibacterial coatings are still in a conceptual and model stage. Numerous challenges remain before these smart antibacterial coatings can be translated into clinical applications.

### 3.1 Development of smart coatings with high bacterial specificity to minimize cytotoxicity

Currently, most smart coatings designed to combat CAUTIs focus on using pH as a stimulus. However, urinary pH may be influenced by diet or other factors, potentially leading to uncontrolled drug release and associated toxicity during use. To minimize cytotoxicity, it is necessary to develop coatings with high specificity to pathogenic bacteria. Beyond single-stimuli responsive systems, such as enzyme-responsive and toxin-responsive coatings, the development of dual-responsive or even multi-responsive coatings may offer a promising strategy to further improve selectivity and responsive speed. In addition, the long-term biocompatibility and safety of these new coatings should also be systematically evaluated *in vivo*.

### 3.2 Long-term antibacterial effectiveness and stability

Currently, most reported smart coatings have been constructed on catheter surfaces using non-covalent functionalization strategy. While this method is relatively simple, it may not provide sufficient stability and long-term antibacterial activity, particularly in the complex microenvironment of the human body. Therefore, the design of covalent and durable responsive coatings is important for developing urinary catheters with long-lasting antibacterial performance. Although some smart coatings for urinary catheters have demonstrated excellent antibacterial performance *in vitro* and *in vivo*, assessments are usually performed over a short period, and the long-term stability of these coatings need to be further assessed.

### 3.3 Establishment of standardized protocols for *in vitro* and *in vivo* tests

**Fig. 16:**
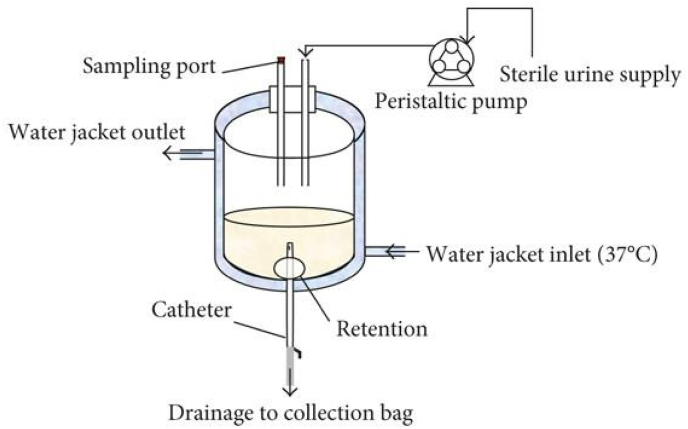
Schematic of an *in vitro* bladder model designed by Stickler et al. [173] Reproduced under terms of the CC-BY license [172]. Copyright 2018, The Authors, published by Wiley-VCH.

Various methods and experimental conditions have been used in antimicrobial tests, but the lack of standardized protocols of *in vitro* antimicrobial tests makes it difficult to compare experimental results and predict the *in vivo* antibacterial effects accurately. In addition to general antimicrobial testing methods (*e.g.* inhibition zone assays, agar plate counting, live/dead staining) which have been reviewed elsewhere [170,171], it is important to use dynamic *in vitro* models to mimic actual flow conditions *in vivo*. When establishing standardized protocols for *in vitro* model testing, several parameters should be considered, including the models, medium, flow conditions, microbial species and strains, and inoculum concentration. Among various *in vitro* urinary tract models [172], the *in vitro* bladder model (Fig. 16) designed by Stickler et al. [173] in 1999 has been widely used and modified. Artificial urine is considered an ideal medium for *in vitro* testing because it reduces individual variability in human urine, yielding more reliable and comparable results. Flow conditions are also important as flow can significantly affect the physicochemical characteristics of biofilms. In detail, urinary flow can affect the imposed shear stress, interactions between planktonic microorganisms and catheter surfaces, as well as the transport of nutrient to the biofilms. Consequently, the hydrodynamic characteristics of medium flow influence biofilm formation and structure, and also affect the tolerance to antimicrobial agents [26,174]. Concerning the selection of microorganisms, it is critical to choose species and strains that can represent a clinical situation. Unfortunately, there are no identified suitable clinically significant model strains for evaluating antimicrobial properties of coatings at this moment [175]. In most studies of smart antibacterial coatings for urinary catheters, *E. coli, P. mirabilis* and *S. aureus* have been widely used as representative bacteria of CAUTIs. However, information about the antimicrobial effect of the developed smart antibacterial coatings to fungal pathogens is lacking in the reported literature. Due to the high infection risk of Candida species during long-term catheterization and the common drug resistance of Candida strains, it is recommended to use Candida albicans as representative fungal pathogen to conduct antimicrobial tests. The ideal range of bacterial density is 10^7^–10^9^ CFU mL^−1^ and for fungal pathogens such as *C. albicans,* 10^5^ CFU mL^−1^ is recommended [175].

Standardization of *in vitro* tests helps to more accurately predict the *in vivo* antimicrobial efficacy, thereby reducing the number of *in vivo* experiments, lowering experimental costs, and minimizing animal suffering. However, *in vivo* tests remain essential before clinical translation, as *in vitro* models cannot fully replicate the complex physiological environment of human body (e.g., the host immune response to implants). At present, there are no standardized *in vivo* models for testing urinary catheters. Various animal models have been used, including rat, rabbit and porcine models. In addition to the choice of animal species, other critical factors including microbial species and strains, inoculation route, and experimental duration should also be carefully considered. More discussion about *in vitro* and *in vivo* models can be found in other literature [175].

### 3.4 Large-scale manufacturing

The manufacturing of smart coatings for urinary catheters is limited to laboratory research due to the complex fabrication process. To endow multiple functions, different functional components need to be integrated into a single system, which usually requires multistep optimization processes, especially when high specificity is required. The large-scale manufacturing of these smart systems may require complex optimization procedures, leading to increased production costs and raising concerns of batch-to-batch reproducibility. Therefore, developing simplified approaches for fabricating smart coatings is essential. For example, designing novel multifunctional components via simple synthesis methods to replace the complex integration of multiple single–functional components into one system.

### 3.5 Regulatory considerations

The last major obstacle to the commercialization of smart antibacterial coatings for urinary catheters is obtaining regulatory approval. Despite the development of smart antibacterial coatings for medical devices in recent years, the clinical translation of these novel technologies has been extremely limited, which can be attributed, in some degree, to strict regulatory approvals. Before bringing new technologies to the market, certification processes from international regulatory bodies, such as the FDA (U.S. Food and Drug Administration) and those responsible for CE marking (Conformité Européenne) in the European Union, requires significant time. Given the complexity of the certification process, it is crucial for researchers and industry to engage proactively with regulatory agencies and healthcare policymakers from the early stages of development. This collaboration helps ensure that the development of smart coatings for urinary catheters aligns with stringent regulatory requirements (e.g., ISO standards, Medical Device Regulation), thereby facilitating smoother approval and accelerating time to market [176].

Overall, the effective prevention, detection and treatment of CAUTIs requires collaborative efforts from clinicians and researchers across various disciplines, including microbiology, materials science, chemistry, and engineering, given the complexity of CAUTIs.

## CRediT authorship contribution statement

**Xiaojin Liu:** Writing – review & editing, Writing – original draft. **Marleen Kamperman:** Writing – review & editing, Supervision.

## Declaration of competing interest

The authors declare that they have no known competing financial interests or personal relationships that could have appeared to influence the work reported in this paper.

## Data Availability

Data will be made available on request.
